# Diagnostic accuracy of ultrasound-based multimodal radiomics modeling for fibrosis detection in chronic kidney disease

**DOI:** 10.1007/s00330-022-09268-3

**Published:** 2022-12-01

**Authors:** Xin-Yue Ge, Zhong-Kai Lan, Qiao-Qing Lan, Hua-Shan Lin, Guo-Dong Wang, Jing Chen

**Affiliations:** 1 Department of Medical Ultrasound, Fourth Affiliated Hospital of Guangxi Medical University, Liuzhou, Guangxi China; 2 Department of Medical Ultrasound, Liuzhou People’s Hospital Affiliated to Guangxi Medical University, Liuzhou, Guangxi China; 3 Department of Radiology, First Affiliated Hospital of Guangxi Medical University, Nanning, Guangxi China; 4 Department of Pharmaceutical Diagnosis, GE Healthcare, Changsha, 410005 China; 5 Department of Oncology, Fourth Affiliated Hospital of Guangxi Medical University, Liuzhou, Guangxi China

**Keywords:** Chronic kidney disease, Ultrasonography, Nomogram, Radiomics, Kidney fibrosis

## Abstract

**Objectives:**

To predict kidney fibrosis in patients with chronic kidney disease using radiomics of two-dimensional ultrasound (B-mode) and Sound Touch Elastography (STE) images in combination with clinical features.

**Methods:**

The Mindray Resona 7 ultrasonic diagnostic apparatus with SC5-1U convex array probe (bandwidth frequency of 1–5 MHz) was used to perform two-dimensional ultrasound and STE software. The severity of cortical tubulointerstitial fibrosis was divided into three grades: mild interstitial fibrosis and tubular atrophy (IFTA), fibrotic area < 25%; moderate IFTA, fibrotic area 26–50%; and severe IFTA, fibrotic area > 50%. After extracting radiomics from B-mode and STE images in these patients, we analyzed two classification schemes: mild versus moderate-to-severe IFTA, and mild-to-moderate versus severe IFTA. A nomogram was constructed based on multiple logistic regression analyses, combining clinical and radiomics. The performance of the nomogram for differentiation was evaluated using receiver operating characteristic (ROC), calibration, and decision curves.

**Results:**

A total of 150 patients undergoing kidney biopsy were enrolled (mild IFTA: *n* = 74; moderate IFTA: *n* = 33; severe IFTA: *n* = 43) and randomized into training (*n* = 105) and validation cohorts (*n* = 45). To differentiate between mild and moderate-to-severe IFTA, a nomogram incorporating STE radiomics, albumin, and estimated glomerular filtration (eGFR) rate achieved an area under the ROC curve (AUC) of 0.91 (95% confidence interval [CI]: 0.85–0.97) and 0.85 (95% CI: 0.77–0.98) in the training and validation cohorts, respectively. Between mild-to-moderate and severe IFTA, the nomogram incorporating B-mode and STE radiomics features, age, and eGFR achieved an AUC of 0.93 (95% CI: 0.89–0.98) and 0.83 (95% CI: 0.70–0.95) in the training and validation cohorts, respectively. Finally, we performed a decision curve analysis and found that the nomogram using both radiomics and clinical features exhibited better predictability than any other model (DeLong test, *p* < 0.05 for the training and validation cohorts).

**Conclusion:**

A nomogram based on two-dimensional ultrasound and STE radiomics and clinical features served as a non-invasive tool capable of differentiating kidney fibrosis of different severities.

**Key Points:**

*• Radiomics calculated based on the ultrasound imaging may be used to predict the severities of kidney fibrosis.*

*• Radiomics may be used to identify clinical features associated with the progression of tubulointerstitial fibrosis in patients with CKD.*

*• Non-invasive ultrasound imaging-based radiomics method with accuracy aids in detecting renal fibrosis with different IFTA severities.*

**Supplementary Information:**

The online version contains supplementary material available at 10.1007/s00330-022-09268-3.

## Introduction

Chronic kidney disease (CKD) describes a state of progressive structural and functional deterioration of the kidney, presenting as a reduced estimated glomerular filtration rate (eGFR). CKD can lead to end-stage kidney disease (ESKD) and is responsible for 9.1% and 4.6% of noncommunicable disease-related morbidity and mortality, respectively [1]. It is projected that CKD will become the fifth leading global cause of death by 2040 [2]. Consequently, timely diagnosis followed by early treatment initiation for those with CKD is crucial for optimizing their outcomes.

Interstitial fibrosis and tubular atrophy (IFTA) are tightly correlated with CKD severity and impact patients’ long-term prognosis. Moderate and severe IFTA, compared to mild IFTA, and global glomerulosclerosis are associated with more than a two- and three-fold increased risk of kidney function loss, respectively [3]. However, current methods for monitoring kidney fibrosis remain unsatisfactory. In clinical practice, eGFR is not always consistent with the degree of renal fibrosis. eGFR can be quite insensitive to subclinical kidney function impairment. Kidney biopsy is considered the gold standard for confirming CKD diagnosis and fibrosis grading [4–6]. However, kidney biopsy carries the risk of complications, and spatial sampling bias reduces the accuracy of pathological diagnosis; therefore, kidney biopsy has not been considered the preferred follow-up approach for patients with CKD [7, 8].

Morphological changes in the kidney cortex and volume mostly occur during ESKD. Under pathological examination, CKD is characterized by kidney fibrosis, or the pathological deposition of massive extracellular matrices related to an increasing number of fibroblasts [9, 10]. These changes are associated with subsequent scarring and sclerosis of kidney tissues, leading to kidney morphological alterations [11]. Ultrasound examinations can assess changes in speckling pattern and signal scattering, both of which variably correlate with changes in kidney morphology and rising parenchymal stiffness. However, distinguishing diseased kidneys from healthy ones using two-dimensional (2D) ultrasound can be difficult for sonographers. These limitations lead to the increased utility of radiomics. Radiomics are quantifications of medical images using statistical algorithms. The machine learning part is used for outcome prediction in subsequent steps. Radiomics aims to support diagnostic decisions through differentiating between different tissue types [12, 13]. Among radiomics, texture analysis is an emerging tool for quantitating the severity of kidney diseases. Radiomics has been applied to different imaging modalities for the identification and differentiation between kidney diseases, including kidney tumors, carcinomas [14–17], the discrimination of malignant and benign clinical T1 renal masses [18] and renal tumor histological subtypes [19], early kidney damage in patients with diabetes mellitus [20], the detection of kidney stones [21], and the differentiation between normal and diseased kidneys in those with CKD [22].

Based on the reasons outlined above, we combined radiomics data from 2D ultrasound and Sound Touch Elastography (STE) images, as well as clinical factors to construct models for application, followed by model verification. We tried to use a nomogram to predict the degree of IFTA among CKD patients without histopathological data. We aimed to provide a non-invasive diagnosis approach for CKD and used this approach to monitor the treatment responses and disease course of these patients.

## Materials and methods

### Ethics statement

The current study complied with the Declaration of Helsinki and was approved by the local ethics review board (KY2021146). We obtained written informed consent from each participant.

### Selection of study participants

The definition of CKD was made based on an eGFR < 60 mL/min/1.73 m^2^ for at least 3 months [23]. The inclusion criteria were CKD patients who had a clinical indication of kidney biopsy. The exclusion criteria were as follows: patients with any contraindications for kidney biopsy, asymmetric bilateral kidney atrophy, abnormal kidney structure, or poor resolution of kidney cortex and medulla on 2D ultrasound. Clinical and laboratory tests were collected from each patient within 2 days before they underwent kidney biopsy.

### Ultrasonography procedures

We used the Mindary Resona 7 Ultrasound System and SC5-1U convex array probe (bandwidth frequency of 1–5 MHz) (Mindray Bio-Medical Electronics Co., Ltd.) to perform 2D ultrasound and STE software. STE measurements were performed 5 times with uniform color fill, and the final standard deviation (SD) of the STE values was set at less than 2.0 as quality control. All examinations were performed by a sonographer with 8 years of experience, who was blinded to serological, imaging, and kidney biopsy pathological results.

### Kidney biopsy and pathological examination

Renal biopsy specimens within 3 days of renal ultrasound were obtained from patients with CKD. A renal needle biopsy was done to sample the lower pole parenchyma of the target kidney under ultrasound (US) guidance. To ensure that the selected US images matched the US biopsy location, the kidney puncture operation, 2D ultrasound, and STE examination were performed by the same sonographer. Two experienced pathologists scored the severity of glomerular sclerosis, tubulointerstitial injury, and vascular sclerosis based on the Banff scoring system and experiences from Farris et al [24, 25]. Any disagreement between pathologists was resolved by consensus. We used the Image-Pro Plus 6.0 software to evaluate the proportion of tubulointerstitial fibrotic areas. Patients with CKD were classified according to the Banff scoring system for kidney cortical fibrosis [26]. In this scoring system, the severity of cortical tubulointerstitial fibrosis was divided into three grades: mild IFTA, fibrotic area < 25%; moderate IFTA, fibrotic area 26–50%; and severe IFTA, fibrotic area > 50%.

### Processing flow of radiomics

#### Image segmentation

Images of Digital Imaging and Communications in Medicine (DICOM) format acquired during B-mode and STE examination were imported into ITK-snap software for manual image segmentation. We evaluated the region of interest (ROI) containing the kidney cortex but removed the kidney medulla and perirenal fat tissues during image curation. Any difference between the two interpreters was resolved by group discussions.

#### Feature extraction and establishment of radiomics label

The DICOM images and ROIs obtained from ITK-SNAP software were imported into the AK software (Artificial Intelligence Kit, GE Healthcare) for extracting radiomics. The extracted features included first-order (histogram and morphologic features) alongside second-order parameters. The second-order parameters mainly involved Gray Level Co-Occurrence Matrix (GLCM), Gray Level Run Length Matrix (GLRLM), Gray Level Size Zone Matrix (GLZSM), Neighboring Gray Tone Difference Matrix (NGTDM), and Gray Level Dependence Matrix (GLDM). The ROI of all images was delineated by two sonographers. The inter-observer agreement was evaluated using interclass correlation coefficient (ICC) analysis, which was defined as good consistency for values between 0.75 and 1, fair consistency for values between 0.4 and 0.75, and poor for values under 0.4. ICC values higher than 0.75 were selected for further analysis. Patients were randomly divided into training and validation cohorts at a ratio of 7:3. We planned for two types of comparisons: mild versus moderate-to-severe IFTA and mild-to-moderate versus severe IFTA.

### Feature selection

Minimum redundancy maximum relevance (mRMR) was used to eliminate redundant and irrelevant features, retain the optimal ones, filter out the optimal feature subset through the least absolute shrinkage and selection operator (LASSO) algorithm, and build a final model. After determining the number of optimal features, we selected the most predictive feature subset and calculated the corresponding coefficients [27].

### Model construction and result validation

Feature extraction based on B-mode and STE images yielded a radiomics quality score (Rad-Score), which was the radiomics label calculated by the weighted summation of selected features by their coefficients. We used receiver operating characteristic curve (ROC) analysis to evaluate the performance of each constructed model. The Akaike information criterion of the clinical model was applied to determine the most appropriate clinical model. Multivariate logistic regression combining clinical features with the Rad-Score was conducted to establish a predictive model and generate a clinical nomogram. The usefulness of a nomogram lies in its ability to map prediction probability to points on a picture with a scale between 0 and 100. The total points accrued based on different types of features corresponded with the predicted probabilities of the index patient [28, 29]

The predictive accuracy of each model was assessed by the area under the ROC curve (AUC) value for the training and validation cohorts. We tested the performance of the Knott diagram in the validation cohort. Using the logistic regression model established in the training cohort, we calculated the total score for each patient in the validation cohort and obtained the AUC and calibration curve. To estimate the prediction error of each model, we further tested the proposed model using the 1000-iteration bootstrap analysis for both the training and validation cohorts. We randomly selected 70% of patients from the training or validation cohort and calculated the corresponding AUC values.

Comparisons between AUCs were made with the DeLong test. The calibration curves and Hosmer–Lemeshow test were used to investigate the performance of the nomogram. Finally, to evaluate the clinical practicability by quantifying the net benefits of the nomogram model in both the training and validation cohorts, the decision curve analysis (DCA) was performed based on clinical features and radiomics labels from B-model, STE-model, B plus STE model, and the combined models. DCA determines the clinical practicability of radiomics nomograms by quantifying the net benefits under different threshold probabilities in the validation set.

### Statistical analyses

SPSS (version 26.0; IBM), GraphPad Prism 8.0 (GraphPad Software), and R statistical software (version 4.0.2) were used for statistical tests. *p* < 0.05 was considered statistically significant.

## Results

### Basic clinical information from participants

**Fig. 1:**
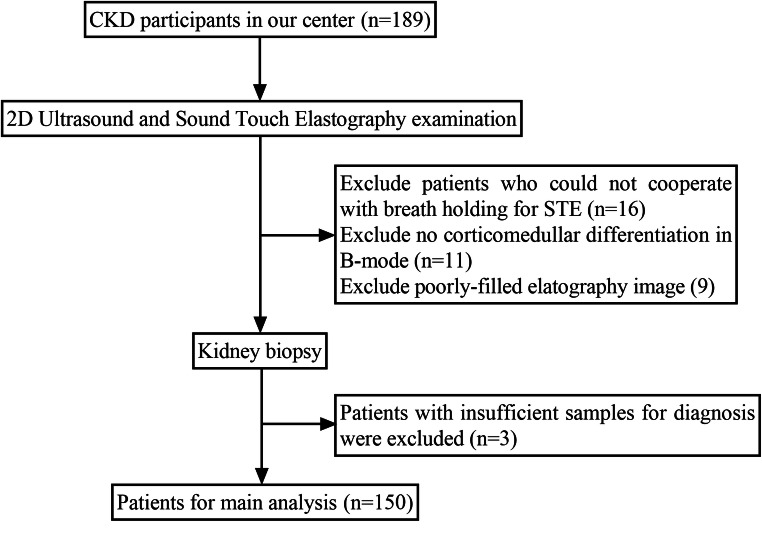
The flowchart of patient selection process

**Fig 2:**
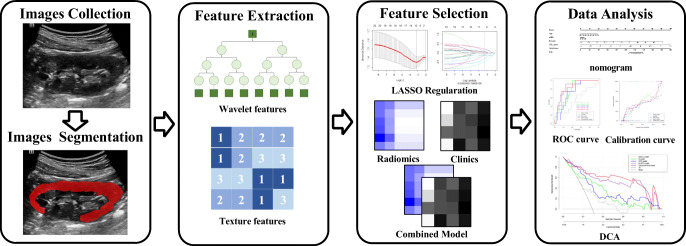
Radiomics flow chart of this study. We exported the collected images in DICOM format, used the ITK software to delineate ROIs, and performed image segmentation. We used the AK software to extract ultrasound radiomics, and built models based on the clinical characteristics of patients with CKD. Later, we performed model calibration and validation.

**Fig. 3:**
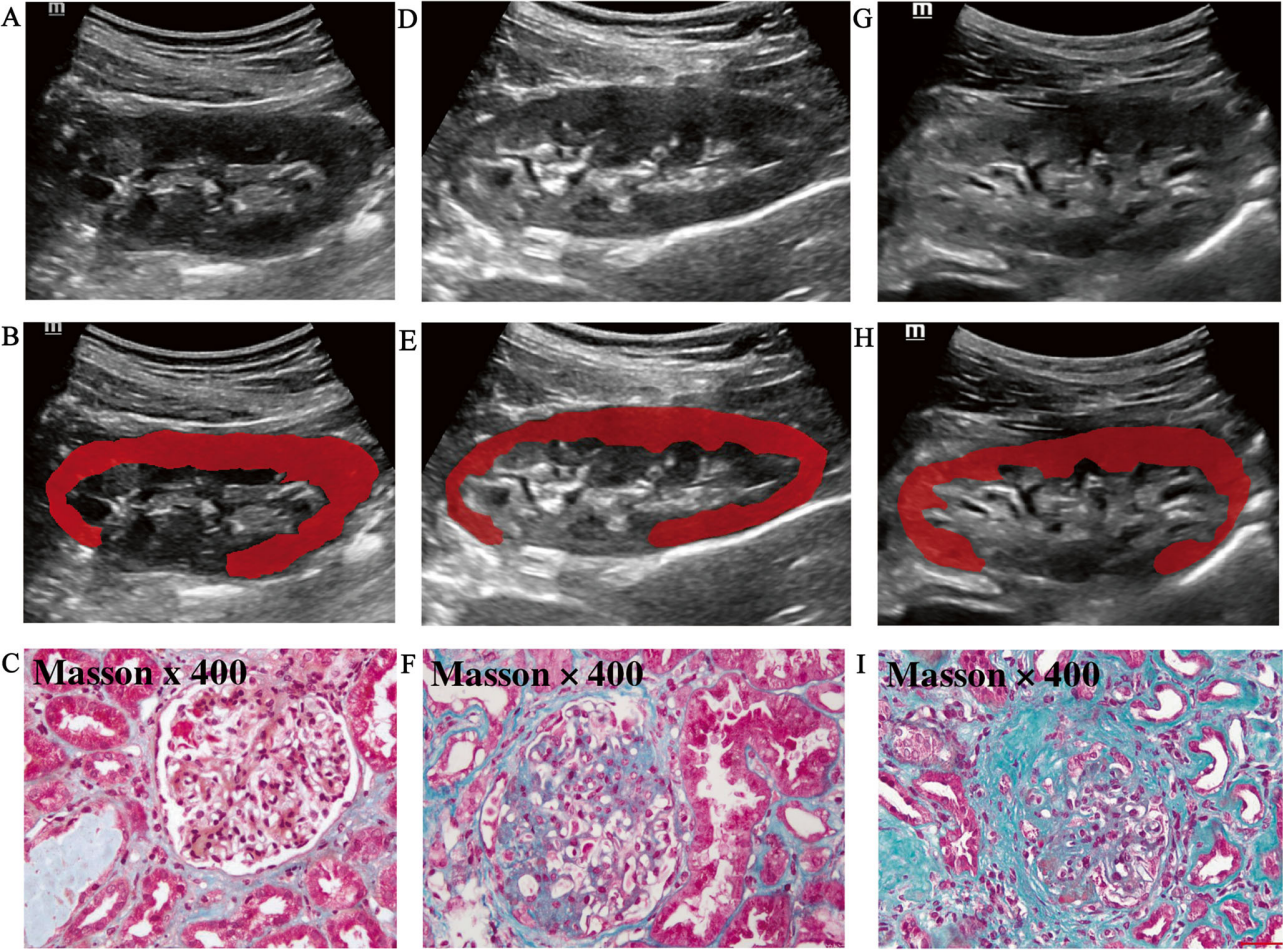
Pathological examinations using Masson staining from kidney biopsy contrasting images from B-mode, and ITK-SNAP ROI in patients with different IFTA severities. **A**–**C** A 38-year-old female patient with systemic lupus erythematosus. Kidney biopsy showing focal proliferative lupus nephritis, III-(A), tubulo-interstitial atrophy was 5% assessed by Masson staining, mild IFTA. **D**–**F** A 36-year-old female patient with chronic kidney disease, 40% tubulo-interstitial atrophy assessed by Masson staining, moderate IFTA; **G**–**I** A 28-year-old male patient with chronic kidney disease. Tubulo-interstitial atrophy assessed by Masson staining was 70%, with severe IFTA

The flowchart of patient selection is provided in Fig. 1. A total of 150 patients with CKD were identified in Table 1, along with their pathological diagnoses (Supplementary Table 1). The course of processing radiomics is shown in Fig. 2. We also illustrated how the ITK-SNAP software delineated the ROI of the punctured kidney cortex (Fig. 3). Table 2 shows the clinical characteristics of training and validation cohorts.

**Table 1:** Patient’s characteristics at baseline

Characteristic	All	Mild IFTA	Moderate IFTA	Severe IFTA	*p* value
No. of patients	150	74	33	43	–
Age (years)	53 (38–64)	50 (33–58)	55 (48–66)	57 (48–67)	0.008
Gender (female)	50 (33.3%)	14 (18.9%)	12 (35.3%)	9 (31.0%)	0.321
BMI (kg/m^2^)	24.27 ± 4.22	24.61 ± 4.2	24.08 ± 4.7	23.83 ± 3.89	0.673
eGFR (mL/min/1.73 m^2^)	48.02 ± 33.70	68.99 ± 30.985	38.49 ± 21.06	19.25 ± 18.12	< 0.001
UACR	268.19 (64.98–593.48)	316.38 (84.1–743.85)	83.4 (29.16–217.29)	311.79 (119.25–546.62)	0.007
Renal length (mm)	96.24 ± 10.75	99.07 ± 8.67	95.76 ± 13.18	91.74 ± 10.58	0.038
Renal width (mm)	46.53 ± 7.38	47.59 ± 8.67	46.39 ± 6.11	44.79 ± 10.39	0.64
Cortical thickness (mm)	11.53 ± 2.41	12.16 ± 2.12	10.91 ± 2.13	10.93 ± 2.82	0.001
ROI depth (mm)	4.50 ± 2.79	4.52 ± 1.22	4.96 ± 1.16	4.13 ± 0.92	0.29
Kidney elasticity (kPa)	13.01 ± 3.41	11.31 ± 2.42	13.85 ± 3.61	15.3 ± 3.19	< 0.001
β2-MG (mg/L)	4.10 (2.45–7.68)	2.87 (2.0–5.11)	4.04 (3.16–6.62)	8.42 (5.1–13.24)	< 0.001
Hemoglobin (g/dL)	112.85 ± 29.69	125.57 ± 28.74	112.15 ± 27.47	91.49 ± 19.06	< 0.001
BUN (mmol/L)	8.10 (5.60–14.40)	6.15 (4.9–8.65)	9.1 (6.6–14.2)	14.3 (9.3–21.3)	< 0.001
Blood albumin (g/L)	33.9 (23.80–39.73)	25.55 (17.95–38.5)	37.9 (32.2–37.9)	35.5 (29.2–38.7)	0.085
Blood glucose (mmol/L)	5.62 (4.79–7.23)	5.36 (4.67–6.76)	5.59 (5.11–8.03)	5.96 (4.79–7.59)	0.107
24h Ualb (mg/d)	1740.90 (506.75–5056.88)	2761.5 (775.8–7703.6)	993.6 (316.95–1926.95)	1879.8 (1183–4002)	0.047

*BMI*, body mass index; *eGFR*, estimated glomerular filtration rate; *UACR*, urinary albumin-to-creatinine ratio; *β2-MG*, β2 microgloulin; *Scr*, serum creatinine; *24h Ualb*, 24h urinary protein; Data are presented as mean ± standard error, median (interquartile range), or count (percentage)

**Table 2:** Clinical characteristics of the training and validation cohorts

Variables	Mild IFTA vs moderate-to-severe IFTA		*p* value	Mild-to-moderate IFTA vs severe IFTA		*p* value
	Training cohort (*n* = 105)	Validation cohort (*n* = 45)		Training cohort (*n* = 105)	Validation cohort (*n* = 45)	
Age (years)	50.2 (17.3)	49.3 (15.7)	0.536	51.1 (17.1)	47.1 (15.9)	0.104
Gender						
Female	35 (33.3)	15 (33.3)	1	34 (32.1)	16 (36.4)	0.61
Male	70 (66.7)	30 (66.7)		72 (67.9)	28 (63.6)	
BMI (kg/m^2^)	23.8 (4.2)	25.4 (4.2)	0.01	24.4 (4.3)	24.1 (3.9)	0.87
eGFR (mL/min/1.73 m^2^)	48.6 (34.6)	46.7 (31.8)	0.85	44 (32.3)	57.7 (35.3)	0.04
Renal length (mm)	96 (10.8)	96.8 (10.7)	0.59	96.1 (10.8)	96.7 (10.7)	0.61
Renal width (mm)	46.6 (7.8)	46.4 (6.4)	0.82	46.6 (7.8)	46.3 (6.2)	0.95
Cortical thickness (mm)	11.5 (2.4)	11.6 (2.5)	0.68	11.5 (2.5)	11.5 (2.3)	0.76
ROI depth (mm)	4.2 (1.1)	5.3 (4.8)	0.042	4.3 (1.2)	5 (4.8)	0.46
Kidney elasticity (kPa)	13.1 (3.4)	12.8 (3.4)	0.51	13.3 (3.6)	12.3 (2.7)	0.21
β2-MG (mg/L)	6.5 (7.8)	6.2 (6.4)	0.69	7 (8.1)	5.1 (5.2)	0.11
Hemoglobin (g/dL)	112.6 (29.9)	113.4 (29.6)	0.88	110.2 (30.8)	119.2 (25.9)	0.06
BUN (mmol/L)	11.4 (8.3)	10.4 (6.6)	0.72	11.9 (8.1)	9.1 (6.9)	0.002
Blood albumin (g/L)	31.4 (9.7)	34.1 (16.8)	0.33	31.3 (10.5)	34.3 (15.8)	0.48
Blood glucose (mmol/L)	6.6 (3.8)	6.8 (3.1)	0.36	6.7 (3.8)	6.6 (2.8)	0.62
24h Ualb (mg/d)	3,255.1 (3,784.2)	3,707 (4,012.9)	0.41	3,583.4 (4,110.3)	2,926.5 (3,113.8)	0.56

### Feature selection, model construction, and results validation

**Fig. 4:**
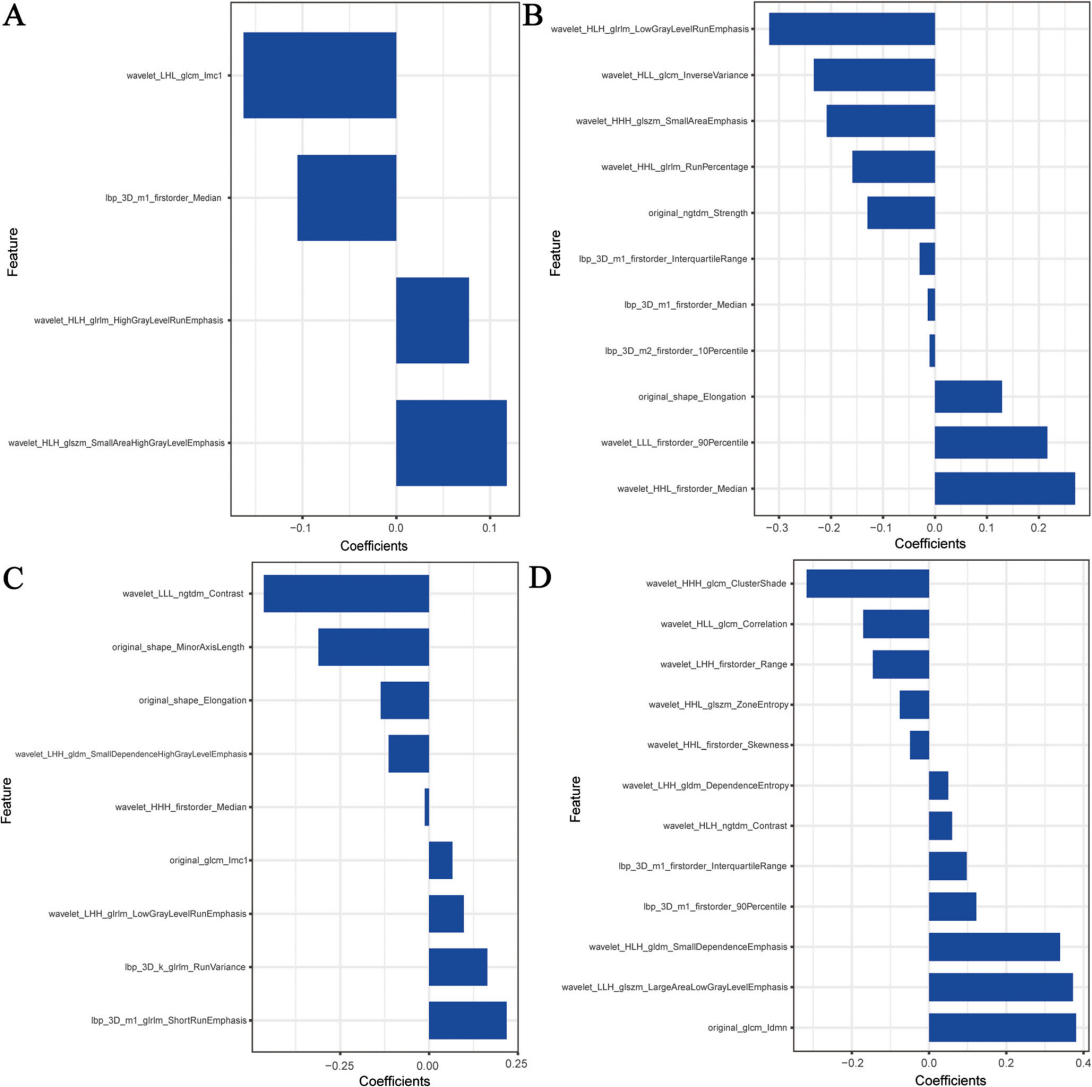
Radiomics signatures for B-mode and STE images. **A** Four features from B-mode images of mild versus moderate-to-severe IFTA; **B** Eleven features from STE images of mild versus moderate-to-severe IFTA; **C** Nine features from B-mode images of mild-to-moderate versus severe IFTA; **D** Twelve features from STE images of mild-to-moderate versus severe IFTA

We extracted 1156 radiomics from the B-mode and STE images for each participant, based on the result of reproducibility analysis by two sonographers, 739 radiomics had good consistency (ICC > 0.75), and retained 120 features after being filtered by the mRMR method. We also did texture feature selection based on the LASSO logistic regression (Supplementary Figure 1) and selected 36 radiomics after the procedure. These features were used to construct the radiomics signature (Fig. 4). The final formula for calculating Rad-Scores is shown in the Supplementary Materials. We compared the Rad-Scores between the training and the testing groups, as shown in Supplementary Figure 2.

We further compared the results of B-model, STE model, clinical model and combined model between groups of different IFTA grades, accompanied by model validation, and prediction parameters were calculated using the Youden index (Table 3). We also showed the results using decision curve evaluation models in Supplementary Figure 3. The predicting models built on clinical features for identifying mild vs. moderate-to-severe IFTA and for mild-to-moderate vs. severe IFTA are shown in Supplementary Figure 4.

**Table 3:** Diagnostic performance of different model prediction for the assessment of IFTA in two training and validation groups

	AUC	ACC	ACC Lower	ACC Upper	Sen	Spe	PPV	NPV
Mild IFTA vs moderate-to-severe IFTA								
B-model								
Train	0.72 (0.63–0.82)	0.67	0.57	0.76	0.55	0.80	0.76	0.61
Test	0.71 (0.55–0.87)	0.71	0.56	0.84	0.75	0.67	0.72	0.70
STE-model								
Train	0.81 (0.73–0.89)	0.75	0.66	0.83	0.95	0.53	0.70	0.90
Test	0.73 (0.58–0.88)	0.69	0.53	0.82	0.83	0.52	0.67	0.73
B-model + STE-model								
Train	0.81 (0.74–0.89)	0.75	0.66	0.83	0.88	0.61	0.72	0.81
Test	0.75 (0.61–0.90)	0.76	0.60	0.87	0.83	0.67	0.74	0.78
Clinical model								
Train	0.88 (0.80–0.96)	0.88	0.80	0.93	0.88	0.88	0.89	0.86
Test	0.80 (0.66–0.94)	0.78	0.63	0.89	0.96	0.57	0.72	0.93
Clinical model+ STE-model								
Train	0.91 (0.85–0.97)	0.85	0.76	0.91	0.79	0.92	0.92	0.79
Test	0.85 (0.73–0.98)	0.73	0.58	0.85	0.58	0.90	0.88	0.66
Mild-to-moderate IFTA vs severe IFTA								
B-model								
Train	0.80 (0.71–0.90)	0.81	0.72	0.88	0.71	0.85	0.67	0.88
Test	0.78 (0.65–0.92)	0.70	0.55	0.83	0.50	0.78	0.46	0.81
STE-model								
Train	0.81 (0.73–0.89)	0.69	0.59	0.78	0.97	0.57	0.48	0.98
Test	0.73 (0.58–0.88)	0.61	0.45	0.76	0.75	0.56	0.39	0.86
B-model + STE-model								
Train	0.93 (0.88–0.98)	0.85	0.77	0.91	0.90	0.83	0.68	0.95
Test	0.86 (0.75–0.97)	0.80	0.65	0.90	0.92	0.75	0.58	0.96
Clinical model								
Train	0.67 (0.55–0.79)	0.68	0.58	0.77	0.68	0.68	0.47	0.84
Test	0.55 (0.34–0.76)	0.75	0.60	0.87	0.25	0.94	0.60	0.77
Clinical model+ B-model + STE-model								
Train	0.93 (0.89–0.98)	0.86	0.78	0.92	0.87	0.85	0.71	0.94
Test	0.83 (0.70–0.95)	0.80	0.65	0.90	0.75	0.81	0.60	0.90

Note: *ACC*, accuracy; *Sen*, sensitivity; *Spe*, specificity; *PPV*, positive predictive value; *NPV*, negative predictive value

### Clinical features combined with ultrasound radiomics model performance and nomogram validation in analyses involving different IFTA group comparisons

**Fig. 5:**
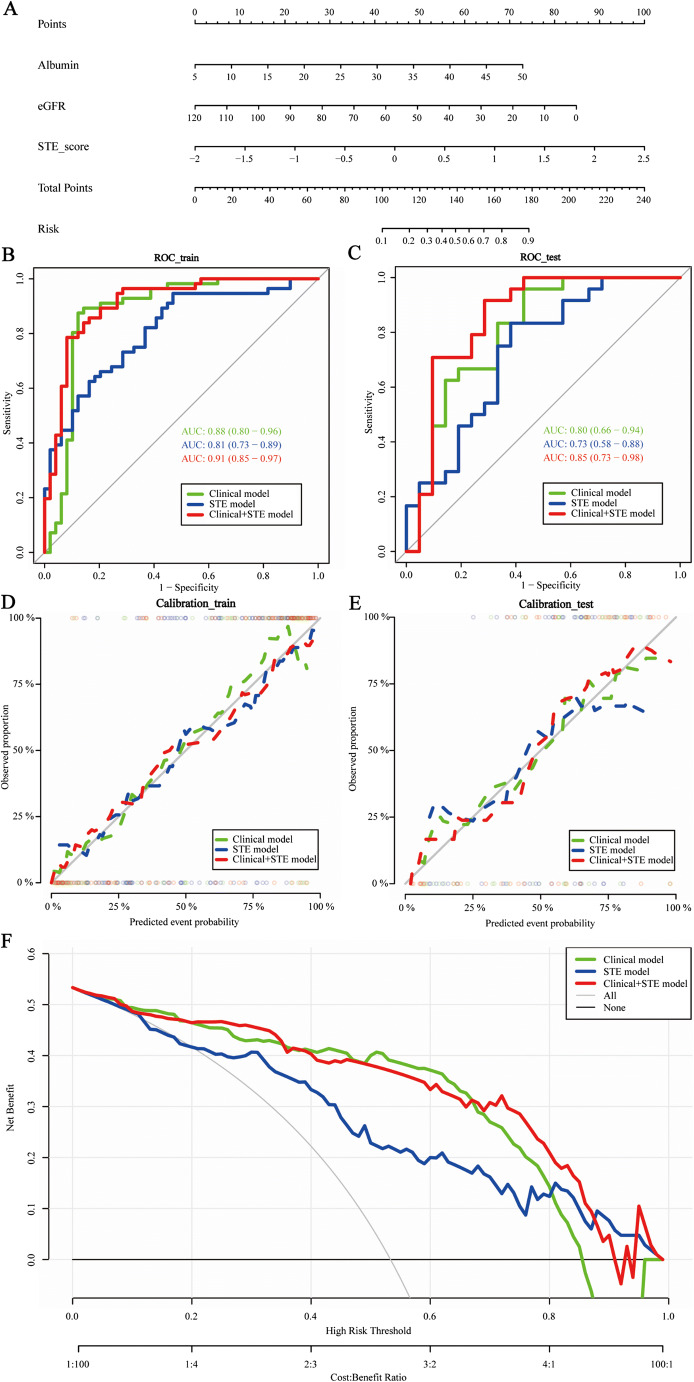
**A** Nomogram for clinical features (albumin and eGFR) of mild vs. moderate-to-severe IFTA combined with STE Rad-Scores. **B, C** Clinical characteristics of mild vs. moderate-to-severe IFTA combined with the ROC curves of STE model in the training and validation sets. **D, E** Calibration curves of the nomogram for clinical model of mild vs. moderate-to-severe IFTA combined with STE model in the training and validation cohorts. **F** Analysis of the cut curve of the histogram for comparison of mild with moderate-to-severe IFTA in the clinical model alone, STE model alone, and combined model. The Y-axis is net income. The blue line represents the decision curve of the STE model. The green line represents the clinical model curve, whereas the red line represents the decision curve of the STE model combined with clinical model of patients with CKD

During the validation of models comparing mild IFTA to moderate-to-severe IFTA, the clinical model established using serum albumin and eGFR achieved moderate prediction ability. Moderate prediction ability was also achieved using the STE radiomics model (Table 3). After adding the results of the STE radiomics model to the clinical model, the predictive performance of the combined model was significantly improved, with the nomogram shown in Fig. 5A, with AUCs of 0.91 (95% CI: 0.85–0.97) and 0.85 (95% CI: 0.77–0.98) for the training cohort and testing cohorts, respectively (DeLong test, *p* < 0.05) (Fig. 5B, C). The nomogram calibration curve showed good agreement between the predictions and observations in the two groups (Fig. 5D, E). The DCA of the nomogram is shown in Fig. 5F. The DCA based on the combined models (clinical and STE) showed greater benefits in the prediction of IFTA severity in the 20–80% threshold probabilities compared to the clinical and STE models.

**Fig. 6:**
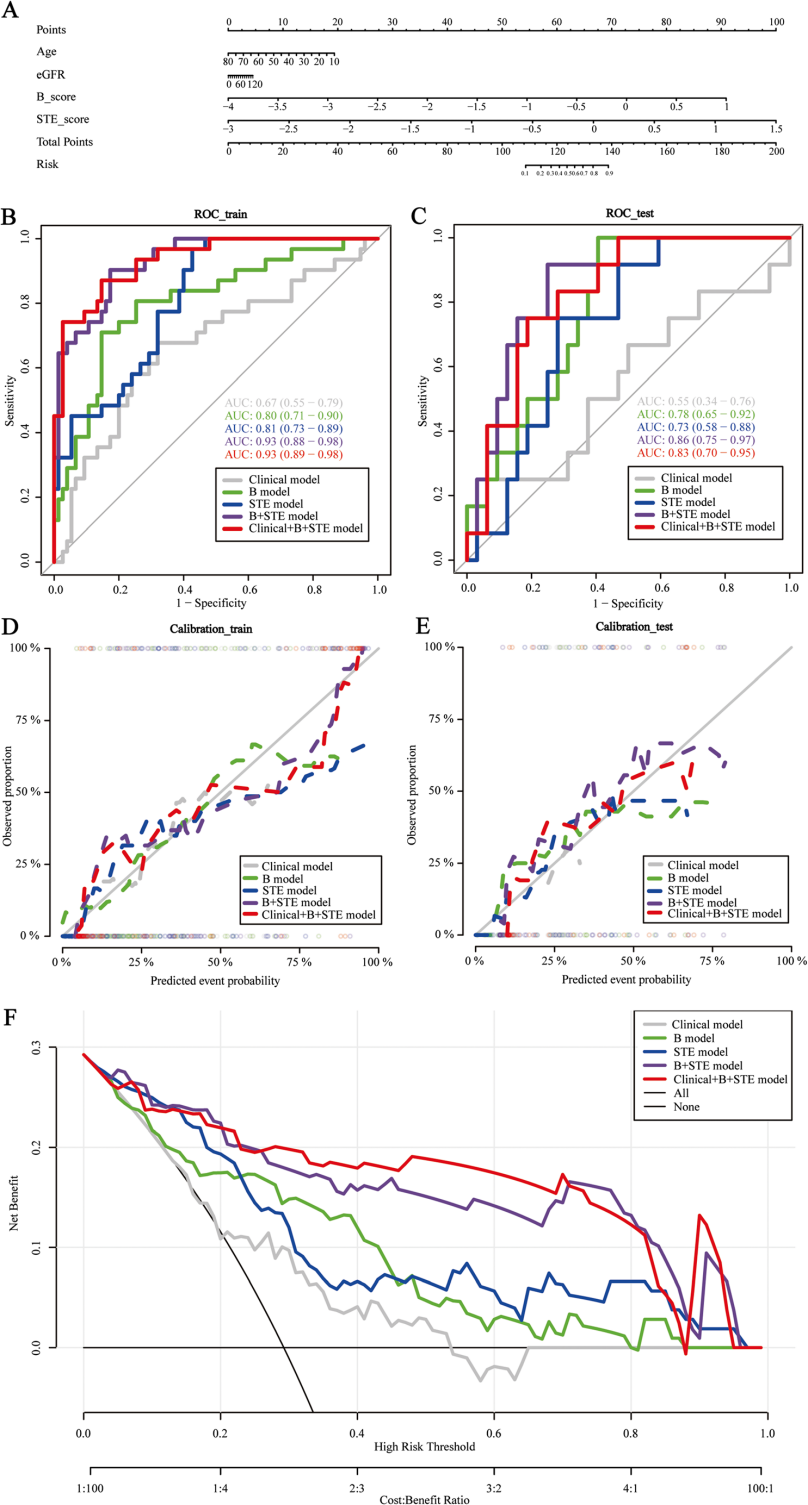
**A** Nomogram for clinical features (age and eGFR) of mild-to-moderate IFTA vs. severe IFTA combined with B-mode Rad-Scores and STE Rad-Scores. **B, C** Clinical characteristics model of mild-to-moderate vs. severe IFTA combined with ROC curves of B-model and STE modelin the training set and validation set. **D, E** Calibration curves of this nomogram for clinical features model of mild-to-moderate vs. severe IFTA in combination with B-model and STE-model in the training and validation cohorts. **F** Analysis of the cutting curve of the nomogram for the clinical model alone, B model alone, STE model alone, and B+STE combined model and the clinical+B+STE combined model comparing mild-to-moderate with severe IFTA. The Y-axis is net income. The gray, green, blue, purple, and red lines represent the clinical model curve, B model curve, decision curve of the STE model, B+STE model curve, and decision curve of the clinical +B+STE model, respectively

During the validation of models comparing mild-to-moderate IFTA to severe IFTA, the clinical model established based on age and eGFR achieved moderate prediction ability, with AUCs of 0.67 (95% CI: 0.55–0.79) and 0.55 (95% CI: 0.34–0.76) for the training and testing cohorts, respectively. Moderate prediction ability was also achieved using the B-mode radiomics model, with AUCs of 0.80 (95% CI: 0.71–0.90) and 0.78 (95% CI: 0.65–0.92) for the training and testing cohorts, respectively. Moderate prediction ability was similarly achieved using the STE radiomics model, with AUCs of 0.81 (95% CI: 0.73–0.89) and 0.73 (95% CI: 0.58–0.88) for the training and testing cohorts, respectively. Higher prediction ability was achieved using the B-mode plus STE radiomics model, with AUCs of 0.93 (95% CI: 0.88–0.98) and 0.86 (95% CI: 0.75–0.97) for the training and testing cohorts, respectively. Finally, models established using age and eGFR, B-mode, and STE radiomics data showed that the prediction ability of the combined model was high, with the nomogram shown in Fig. 6A. The AUCs of the training and the testing cohorts were 0.93 (95% CI: 0.89–0.98) and 0.83 (95% CI: 0.70–0.95), respectively (Fig. 6B, C). The AUCs of the combined model significantly differed from those of the clinical model, B model, or STE model (DeLong test, *p* < 0.005 for the training and validation cohorts). The nomogram calibration curves showed good agreement between predictions and observations in the two groups (Fig. 6D, E). The DCA of the nomogram is shown in Fig. 6F. Compared to other models, the combined nomogram model, showing the highest area under the curve, is the optimal decision making for maximal net benefit in classifying IFTA severity.

## Discussion

The accurate and non-invasive classification of kidney fibrosis severities is crucial for clinical practice. Recently, researchers used machine learning based on elastography ultrasound images to gauge the severity of kidney fibrosis, with promising results [30]. The construction of a binary classification model is mostly used for comparing liver and kidney fibrosis severities [30–33]. In this study, we performed a binary classification by comparing one IFTA grade with the other grades as an approach. A combined model incorporating 2D ultrasound radiomics, STE radiomics, and clinical features for predicting IFTA severities was constructed and validated.

Among clinical features analyzed in this study, eGFR was an independent parameter as shown in different IFTA prediction models (Figs. 5 and 6), consistent with results from Zhu et al [30]. eGFR is an important indicator for estimating kidney function and assessing IFTA severity in patients with CKD [34]. eGFR is calculated based on a standardized formula using Scr, a laboratory index that is widely used for the clinical follow-up of these patients [35]. In the comparative model of mild and moderate-to-severe IFTA, the combined model based on clinical features (serum albumin and eGFR) and STE radiomics further improved the diagnostic performance. eGFR has limitations as an indicator for kidney fibrosis, since the levels of eGFR are frequently inconsistent with the degree of kidney fibrosis. Furthermore, eGFR is not sensitive to subclinical kidney damage [36]. In our training and testing cohorts, comparing mild-to-moderate IFTA to severe IFTA, the AUC of the clinical features (eGFR and age) model for discrimination was 0.67 (95% CI: 0.55–0.79) and 0.55 (95% CI: 0.34–0.76) in the training and testing cohorts, respectively, suggesting that clinical features model only performed worse than B-model or STE-model only or the combined model (Fig. 6B, C). Judging from the above arguments, we selected IFTA severity as the grouping variable and prediction model construction instead of eGFR. Therefore, a combined model consisting of clinical factors of eGFR and ultrasonography radiomics features can be helpful for achieving non-invasive monitoring of kidney fibrosis.

The main factors affecting STE elasticity measurements are anisotropy and the heterogeneity of kidney fibrosis. Other confounding factors for STE measurement include age and BMI [30]. In this study, age was used to construct a nomogram for predicting the comparison between mild and moderate-to-severe IFTA. Clinical model established by age and eGFR, and the combined model all achieved a fair predictive performance. In reality, kidneys become stiffened due to collagen deposition during ESKD, and STE measurement results will increase. However, with renal function further declining, kidneys may become softer due to poor blood perfusion, and the STE measurement results may decrease, whereas the kidney length becomes smaller on 2D ultrasound examination [37–39]. These factors likely lead to the emergence of a complex nonlinear relationship between 2D ultrasound measurements, STE measurements, and IFTA severity. In our study, a combined model built based on B-mode and STE results significantly improved the diagnostic performance of traditional ultrasound alone. Possible explanations for this finding include the ability of STE to capture the stiffness feature of patients’ kidneys, which is suitable for application during machine learning whose strength includes combining variables with nonlinear relationships and interactions [40]. Therefore, we used all variables including 2D ultrasound, STE radiomics, and clinical factors from these patients with CKD to model IFTA.

In the nomogram differentiating mild and moderate-to-severe IFTA models, STE radiomics and clinical factors were included, whereas 2D ultrasound radiomics were not. The reason is that the 2D ultrasound radiomics consist of data including the diameter of the kidney’s long axis and its cortical thickness. However, in patients with mild and moderate IFTA, changes in their kidney morphology remain minimal due to their early CKD stages [41]. In this study, there were no differences in kidney lengths and cortical thickness between different IFTA groups (*p* = 0.487 and *p* = 0.927 for the mild and moderate IFTA groups, respectively). During our construction of a comparison model between the mild and moderate-to-severe IFTA groups, we extracted 2D ultrasound image features from those with moderate-to-severe IFTA. Since the radiomics of moderate IFTA were included, the kidney morphological features that did not significantly differ between those with mild and moderate IFTA were extracted. The presence of redundant information might increase the probability of model overfitting, reducing model performance after constructing a joint model. However, when we compared between those with mild-to-moderate and severe IFTA, patients with ESKD and severe IFTA were more likely to have morphological kidney atrophy and cortical thinning. When we compared renal long-axis diameter and cortical thickness between severe and mild-to-moderate IFTA groups, there were differences between groups (*p* < 0.01). Therefore, the addition of radiomics including morphological differences of the kidneys in 2D ultrasound greatly increased the diagnostic performance of the combined model.

The combined model incorporating B-mode, STE, and clinical features can be applicable for IFTA detection for patients outside our training cohort, particularly during the follow-up of patients unable to receive a renal biopsy. The establishment of ultrasound radiomics model can be a great support for clinical ultrasound practice, and radiomics findings may assist in IFTA prediction in the future.

This study has some limitations. Patients selected were those with CKD and renal biopsy indications. The renal cortical tissues of patients with ESKD could be thin, precluding the derivation of histopathological results based on renal biopsy. The sample size of patients with severe IFTA was small, necessitating further expansion to reduce data redundancy during model construction, in order to facilitate the establishment of multi-classification models. In addition, this study was done based on data from one center, using a single-mode ultrasound diagnostic apparatus to collect ultrasound radiomics. Multi-center and different ultrasound modes may be needed to extract more 2D and ultrasound elasticity radiomics to construct a combined model and to test the generalizability of our established combined model. Finally, changes in 2D ultrasound and STE features and the course of CKD among these patients need to be further monitored and validated in the future.

## Conclusion

STE combined with 2D ultrasound examinations can improve the diagnostic performance of traditional ultrasound for tubulointerstitial fibrosis in patients with CKD. The radiomics nomograms constructed based on 2D ultrasound and STE imaging features in combination with clinical features are non-invasive tools with high accuracy in detecting renal fibrosis with different IFTA severities. This approach can be helpful for non-invasive monitoring of kidney fibrosis.

## Supplementary Information

ESM 1 (DOCX 1.35 mb)

## Abbreviations

AUC: Area under the curve
CKD: Chronic kidney disease
DCA: Decision curve analysis
eGFR: Estimated glomerular filtration rate
ESKD: End-stage kidney disease
IFTA: Interstitial fibrosis and tubular atrophy
LASSO: Least absolute shrinkage and selection operator
mRMR: minimum Redundancy Maximum Relevance
NPV: Negative-predictive value
PPV: Positive-predictive value
ROC: Receiver operating characteristic
ROI: Region of interest
Scr: Serum creatinine
STE: Sound Touch Elastography
UPCR: Urinary protein-to-creatinine ratio

## Declarations

### Guarantor

The scientific guarantor of this publication is Xin-Yue Ge.

### Statistics and biometry

One of the authors has significant statistical expertise.

### Methodology

• retrospective

• diagnostic or prognostic study

• performed at one institution
