# Supplementary material for: Diagnostic accuracy of ultrasound-based multimodal radiomics modeling for fibrosis detection in chronic kidney disease
Source: Eur Radiol. 2022 Dec 1;33(4):2386–98. doi: 10.1007/s00330-022-09268-3 (PMC10017610; doi:10.1007/s00330-022-09268-3)
Supplement: Supplementary file 1 — (DOCX 1.35 mb) [file 330_2022_9268_MOESM1_ESM.docx]

Supplementary Table 1: Pathological diagnosis of patients with CKD.

| **Etiology** | N |
| --- | --- |
| IgA Nephropathy | 36 |
| Diabetic Nephropathy | 28 |
| Membranous Nephropathy | 26 |
| Minimal Change Disease and Focal Segmental Glomerulosclerosis | 21 |
| Hypertensive nephrosclerosis | 11 |
| Lupus Nephritis | 7 |
| Amyloidosis nephropathy | 7 |
| Crescentic Glomerulonephritis | 6 |
| Tubulointerstitial Diseases | 6 |
| Henoch-Schönlein Purpura nephritis | 1 |
| Postinfectious Glomerulonephritis | 1 |

In the LASSO model, the penalty parameter λ is chosen using 10-fold cross-validation as the minimum criterion. log(λ) (x-axis) corresponds to partial likelihood deviation (y-axis). The minimum criterion and the 1-SE criterion are used to draw the dashed line at the optimal value. For 57 texture features, LASSO coefficient profiles are displayed. 10-fold cross-validation of log (λ) series was used to draw vertical lines for selected values; 10 non-zero coefficient features were also indicated.


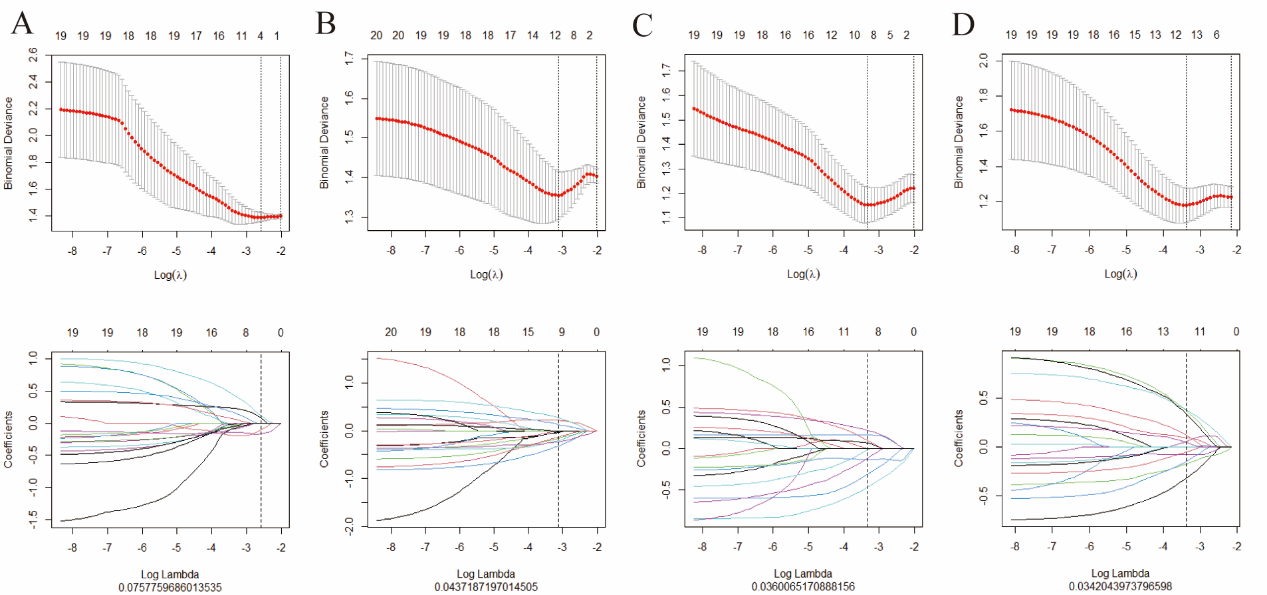


**Supplementary Figure 1** LASSO was used to screen the image omics features, and the10-fold cross validation method was used in LASSO to screen the feature sets with the best performance. A: B-model based imaging feature screening for mild and moderate-to-severe IFTA staging. B: Radiomics feature screening based on STE for mild and moderate-to-severe IFTA staging. C: B-model based imaging feature screening for mild-to-moderate and severe IFTA staging. D: Radiomics feature screening based on STE for mild-to-moderate and severe IFTA staging.

The final formula for calculating radscores.

1. **Radiomics radscore formula for mild and moderate-to-severe IFTA based on B-mode images:**

Radscore=-.105*lbp_3D_m1_firstorder_Median+0.118*wavelet_HLH_glszm_SmallAreaHighGrayLevelEmphasis+-.163*wavelet_LHL_glcm_Imc1+0.078*wavelet_HLH_glrlm_HighGrayLevelRunEmphasis + 0.137。

2. **Radiomics radscore formula for mild and moderate-to-severe IFTA based on STE-mode images:**

Radscore=-.029*lbp_3D_m1_firstorder_InterquartileRange+-.014*lbp_3D_m1_firstorder_Median+-.01*lbp_3D_m2_firstorder_10Percentile+0.216*wavelet_LLL_firstorder_90Percentile+-.159*wavelet_HHL_glrlm_RunPercentage+-.319*wavelet_HLH_glrlm_LowGrayLevelRunEmphasis+-.208*wavelet_HHH_glszm_SmallAreaEmphasis+0.129*original_shape_Elongation+0.269*wavelet_HHL_firstorder_Median+-.233*wavelet_HLL_glcm_InverseVariance+-.13*original_ngtdm_Strength + 0.15。

3. **Radiomics radscore formula for mild-to-moderate and severe IFTA based on B-mode images:**

Radscore=-.136*original_shape_Elongation+-.465*wavelet_LLL_ngtdm_Contrast+0.066*original_glcm_Imc1+0.098*wavelet_LHH_glrlm_LowGrayLevelRunEmphasis+0.164*lbp_3D_k_glrlm_RunVariance+-.012*wavelet_HHH_firstorder_Median+0.218*lbp_3D_m1_glrlm_ShortRunEmphasis+-.311*original_shape_MinorAxisLength+-.114*wavelet_LHH_gldm_SmallDependenceHighGrayLevelEmphasis + -1.014。

4. **Radiomics radscore formula for mild-to-moderate and severe IFTA based on STE-mode images:**

Radscore=0.097*lbp_3D_m1_firstorder_InterquartileRange+0.381*original_glcm_Idmn+0.06*wavelet_HLH_ngtdm_Contrast+0.122*lbp_3D_m1_firstorder_90Percentile+-.171*wavelet_HLL_glcm_Correlation+-.076*wavelet_HHL_glszm_ZoneEntropy+-.317*wavelet_HHH_glcm_ClusterShade+-.049*wavelet_HHL_firstorder_Skewness+0.372*wavelet_LLH_glszm_LargeAreaLowGrayLevelEmphasis+-.146*wavelet_LHH_firstorder_Range+0.049*wavelet_LHH_gldm_DependenceEntropy+0.339*wavelet_HLH_gldm_SmallDependenceEmphasis + -1.007。


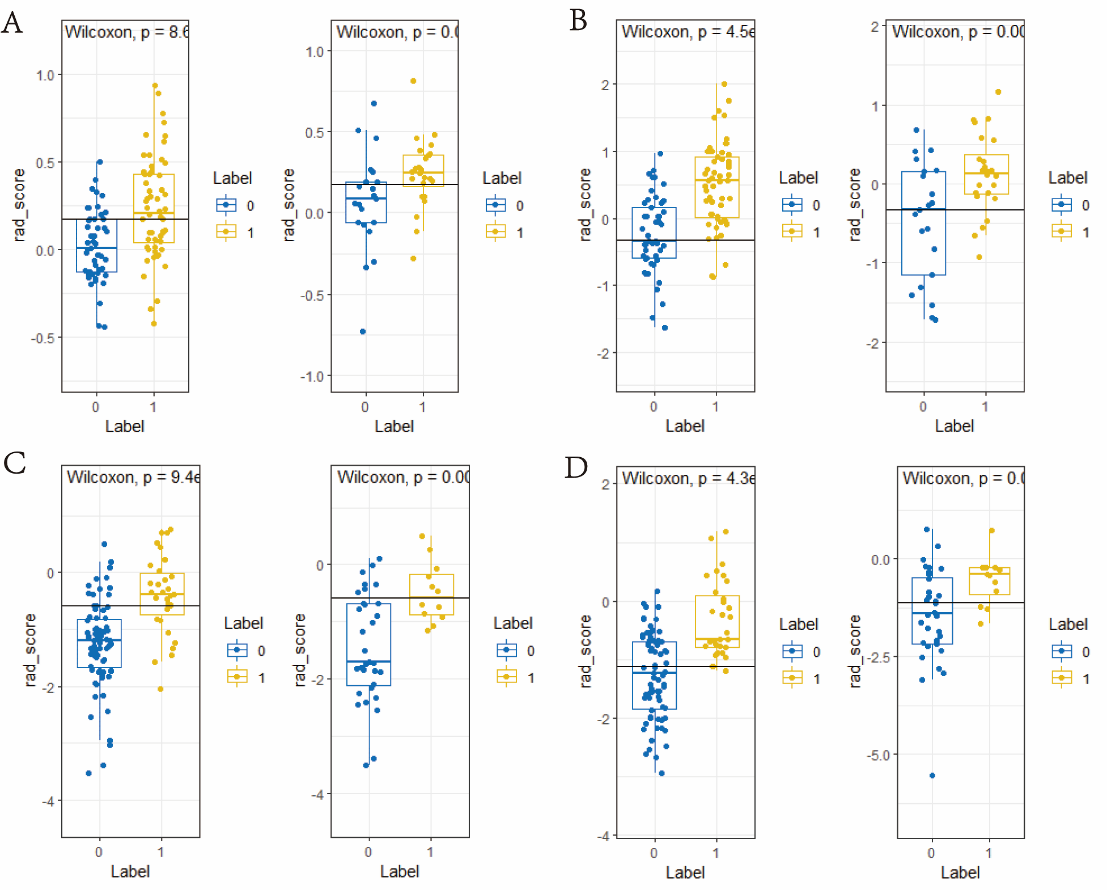


**Supplementary Figure 2** Compare the radscore of different IFTA graded training and test groups respectively. A: Rad-Score comparison between the training group and the test group for mild and moderate-to-severe IFTA staging based on B-model. B: Rad-score comparison between the training group and the test group of mild and moderate-to-severe IFTA staging based on STE. C: Comparison of Rad-score between the training group and the test group for mild-to-moderate and severe IFTA staging based on B-model. D: Rad-score comparison between the training group and the test group for mild-to-moderate and severe IFTA staging based on STE.


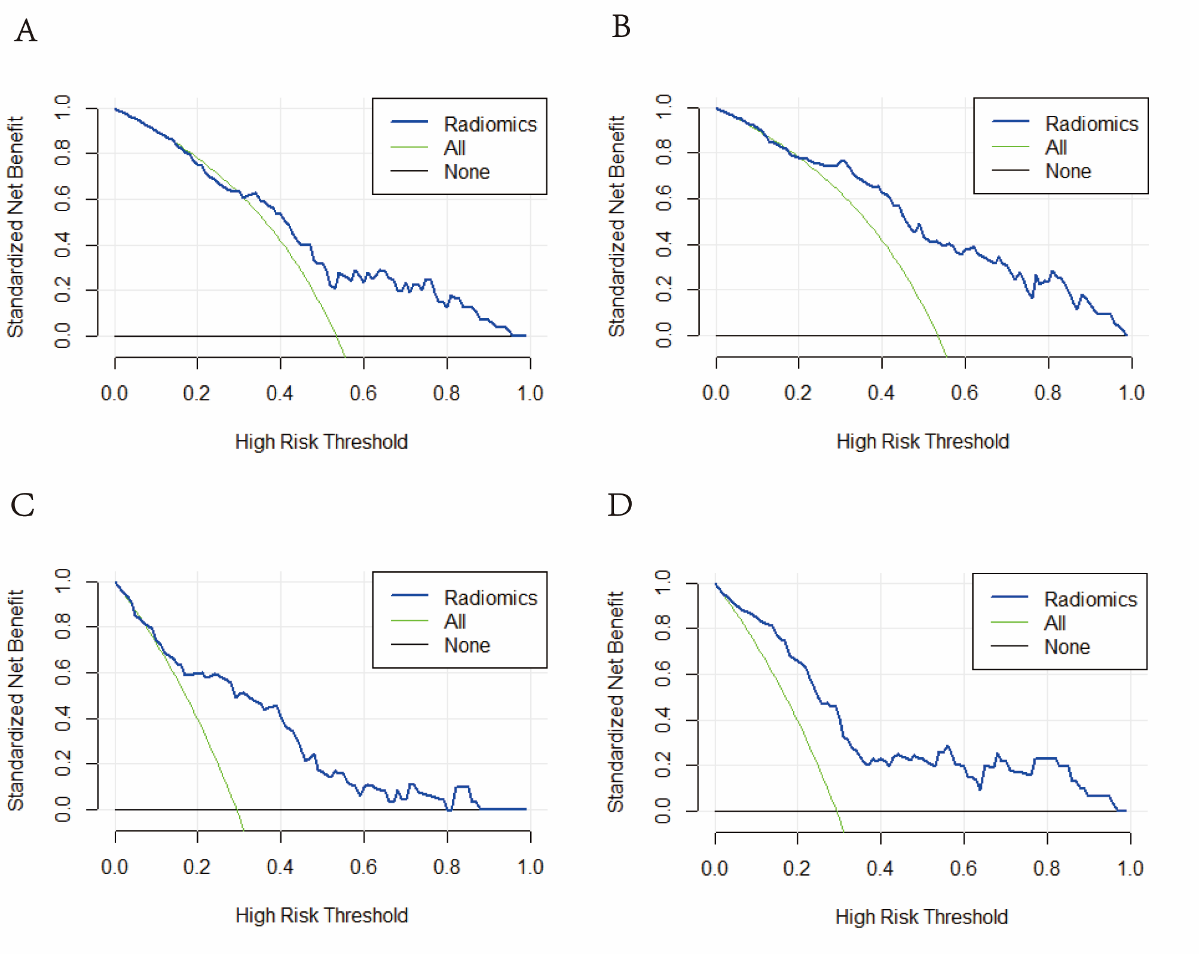


**Supplementary Figure 3** Decision curve analysis of image omics line graph. The Y-axis measures the net benefit. The blue line represents the decision curve of the image omics model. A: In the B-mode of mild and moderate-to-severe IFTA. B: In STE models of mild and moderate severe IFTA. C: In the B-model model of mild-to-moderate and severe IFTA. D: In STE models of mild-to-moderate and severe IFTA.


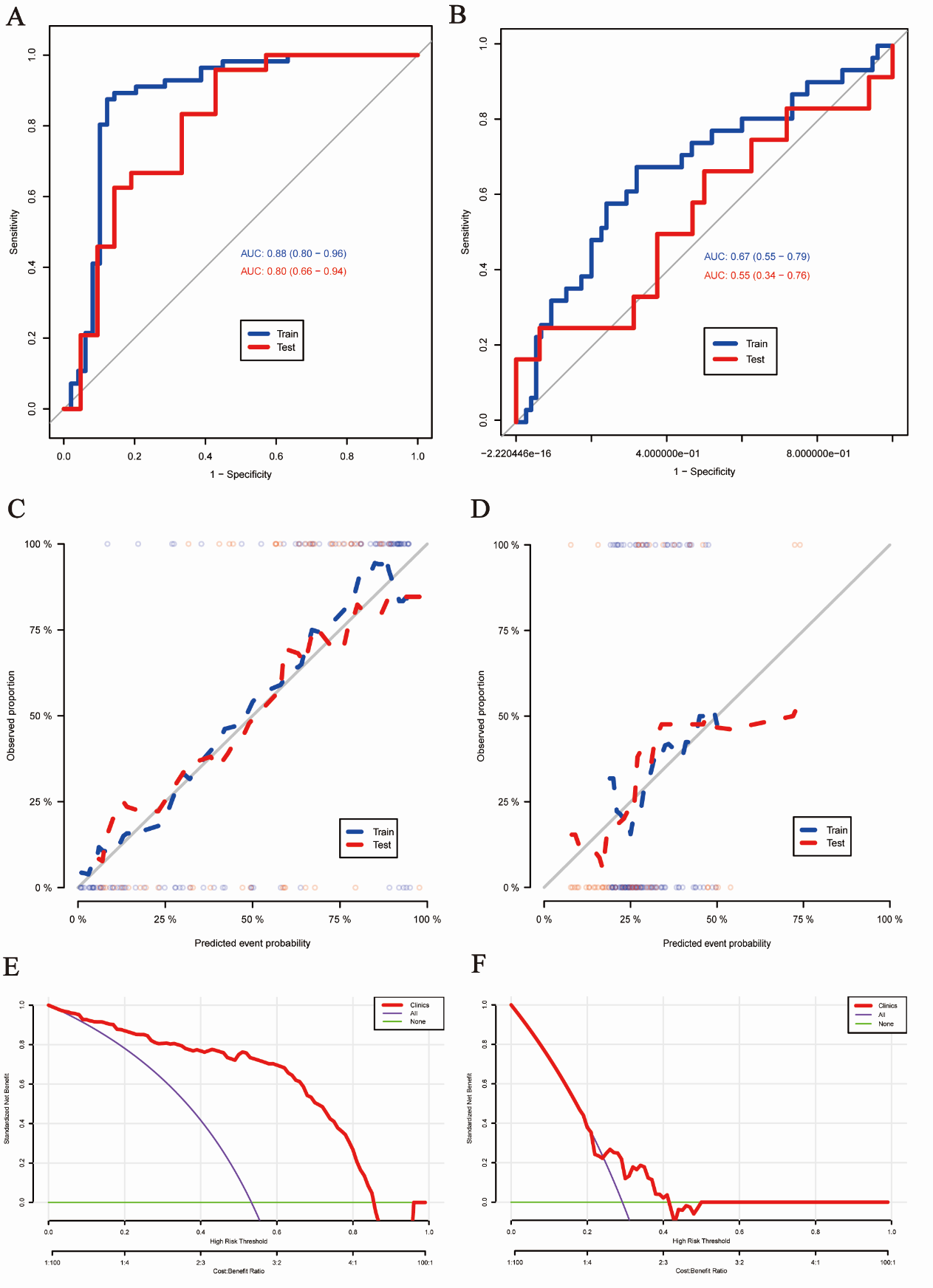


**Supplementary Figure 4A-B** clinical characteristics of mild and moderate-to-severe IFTA as well as mild-to-moderate and severe IFTA groups were modulated in the training set and validation set subject operating characteristic curves. **C-D** Calibration curves of nomogram for clinical features in training and validation cohorts. **E-F:** Decision curve analysis of clinical feature nomogram. The Y-axis is net income. The red line represents the decision curve of the image omics model.
